# Anti-inflammatory effects of the root, stem and leaf extracts of *Chloranthus serratus* on adjuvant-induced arthritis in rats

**DOI:** 10.1080/13880209.2020.1767159

**Published:** 2020-06-05

**Authors:** Shuping Sun, Shengli Li, Yunyan Du, Chenguang Wu, Mengyuan Zhang, Jiarong Li, Xiaoping Zhang

**Affiliations:** a College of Pharmacy, Wannan Medical College, Wuhu, Anhui, China; b College of Life Science, Anhui Normal University, Wuhu, Anhui, China; c Institute of Natural Daily Chemistry, Wannan Medical College, Wuhu, Anhui, China; d The Fifth People’s Hospital of Wuhu, Wuhu, Anhui, China

**Keywords:** Rheumatoid arthritis, complete Freund’s adjuvant, pro-inflammatory mediators, oxidative stress, articular cartilage, arthritic score

## Abstract

**Context:**

*Chloranthus serratus* [(Thunb.) Roem. et Schult, (Chloranthaceae)] is a folk medicine used for the treatment of rheumatoid arthritis.

**Objective:**

The aim of this study was to investigate anti-arthritic effects of the ethanol extracts of the roots (ER), stems (ES) and leaves (EL) of *C. serratus* on adjuvant arthritis rats and related mechanisms.

**Materials and methods:**

The rats were immunized by intradermal injection of complete Freund’s adjuvant (CFA, 0.18 mL) into the right hind feet, and received intragastric administrations of the ER, ES and EL (2.07, 1.61 and 0.58 g/kg/d, respectively) for 14 days. The anti-arthritic activity was assessed by swelling rates, serum indicators, antioxidant capacity, histopathological and immunohistochemical analyses.

**Results:**

The LD_50_ of the ER, ES and EL was higher than 10.35, 8.05 and 2.90 g/kg/p.o., respectively. Extract treatments decreased swelling rates, tumour necrosis factor-alpha (TNF-α), vascular endothelial growth factor (VEGF), interleukin 1 beta (IL-1β), migration inhibitory factor 1 (MIF-1), immunoglobulin G (IgG) and immunoglobulin M (IgM) levels and positive expression of VEGF in the arthritic rats (*p* < 0.01 or *p* < 0.05). The ER significantly decreased NO (3.91 ± 0.61 µmol/L), IL-6 (75.67 ± 16.83 pg/mL) and malondialdehyde (MDA) (2.28 ± 0.32 nmol/mL) contents and clearly increased IFN-γ (2082 ± 220.93 pg/mL) and superoxide dismutase (SOD) (601.98 ± 38.40 U/mL) levels. The ES and EL did not reverse the changes in some indicators. All the extracts alleviated inflammatory cell infiltration and synovial cell proliferation. Among them, the ER was the most pronounced.

**Discussion and conclusions:**

ER exerts the most promising effects, as shown by inhibiting the releases of inflammatory cytokines and enhancing antioxidant capacity, which provides a scientific basis for further research on *C. serratus* and its clinical applications.

## Introduction

Rheumatoid arthritis (RA) is a chronic autoimmune inflammatory disease that manifests as inflammatory synovitis, multiple and invasive arthritis, and cartilage and bone erosion, which eventually leads to articular destruction and deformity (Stump et al. 2011). In addition, RA has a high disability rate, which seriously affects the quality of patients’ life (Stump et al. 2011). Previous studies have shown that the immune system and inflammatory response regulated by pro-inflammatory and anti-inflammatory cytokines play important roles in the pathogenesis and progression of RA (Hassana et al. 2019).

At present, the main drugs used to treat RA in the domestic market are divided into three categories: nonsteroidal anti-inflammatory drugs such as acetylsalicylic acid, steroidal anti-inflammatory drugs such as glucocorticoids, and immunosuppressive drugs, mainly methotrexate (Khaled et al. 2010). Although these drugs can control the symptoms of RA to a certain extent, it is difficult to achieve ideal therapeutic effects on patients with RA due to the differences in the individual tolerances and various toxicities and side reactions of the drugs, which limit their wide applications (Wang et al. 2018). In addition, many traditional Chinese medicines (TCMs) have good anti-inflammatory and immunosuppressive activities (Rocha et al. 2013). TCMs generally exert regulatory effects and show unique multicomponent, multilevel and multitarget advantages in the treatment of RA, thus attracting increasing attention (Qiao et al. 2013). Therefore, the development of new anti-RA drugs with good safety, high efficiency and wide applicability has become a research hotspot in this field.

*Chloranthus serratus* [(Thunb.) Roem. et Schult (Chloranthaceae)] is a perennial herb, whose roots or whole herbs usually are used as a medicine. This herb is mainly produced in Anhui, Zhejiang, Guangxi, Yunnan and other provinces in China. The *Modern Chinese Medicine Dictionary* reports that *C. serratus* promotes blood circulation, relieves phlegm and pain, and treats rheumatic joint pain (Song et al. 2001). Its anti-inflammatory effect is mainly attributed to its terpenoid components (Tang et al. 2016; Li et al. 2018). Previous experiments have shown that the ethanol extract of *C. serratus* roots has obvious anti-inflammatory activity (Sun and Li 2013). Moreover, the water-extractable components of *C. serratus* might exert an anti-inflammatory effect by inhibiting pro-inflammatory cytokines and mediators in lipopolysaccharide-stimulated macrophages (Sun et al. 2019). The evaluation of the therapeutic effect on arthritis *in vivo* has not been reported.

Complete Freund’s adjuvant (CFA)-induced arthritis, a type of chronic inflammation, involves multiple systemic changes, including synovial hyperplasia, inflammatory cell infiltration and abnormal increases in the levels of many cytokines [particularly interleukin 1 beta (IL-1β), tumour necrosis factor-alpha (TNF-α), interleukin 6 (IL-6) and vascular endothelial growth factor (VEGF)], as well as consequent cartilage and bone destruction characterized by swelling, deformation and loss of joint function (Mbiantcha et al. 2017). However, a detailed comparative study on the anti-arthritic effects of the ethanol extracts of *C. serratus* roots, stems and leaves on CFA-induced RA rats has not been performed. In this study, we compared the anti-arthritic activity of the ethanol extracts of *C. serratus* roots, stems and leaves in CFA-induced arthritis rats to identify which extract exerts the most robust anti-arthritic effects. These results will lay a foundation for further study on the anti-arthritic effects of the *C. serratus* extracts and may contribute to the development of more effective treatment strategies for RA.

## Materials and methods

### Animals and ethics statement

Male Sprague–Dawley (SD) rats (200 ± 10 g) aged 6 weeks were procured from Shandong Experimental Animal Center [SCXK (Lu) 2018-0013]. These rats were housed under controlled conditions of light (12 h light/dark cycle), temperature (20–25 °C) and humidity (50 ± 5%) with free access to standard pellet diet and water. The rats were allowed to acclimatize to the feeding conditions for 1 week before the experiment. All experimental procedures were performed in accordance with the guidelines of the Animal Control and Supervision Committee. The Ethics Committee of Wannan Medical College reviewed all animal experimental procedures and approved the use of the experimental animals (WNMC No. 20180316).

### Reagents

The ELISA kits for IL-6, interferon gamma (IFN-γ), immunoglobulin M (IgM), immunoglobulin G (IgG), migration inhibitory factor 1(MIF-1), VEGF, TNF-α and IL-1β used in this study were purchased from Shanghai Youchu Trading Co., Ltd. (Shanghai, China). Superoxide dismutase (SOD), nitric oxide (NO) and malondialdehyde (MDA) kits were obtained from Nanjing Jiancheng Technology Co., Ltd. (Nanjing, China). Rabbit anti-VEGF antibody and immediate SABC-POD (rabbit IgG) kits were supplied by Boster Biotechnology Co., Ltd. (Wuhan, China).

### Plant materials, extract preparation and UV fingerprint analysis

*Chloranthus serratus* was purchased from Yulin Chinese Medicine Port (Guangxi, China). It was identified to be genuine by Professor Jianhua Zhu of Wannan Medical College according to the *Modern Chinese Medicine Dictionary* (Song et al. 2001). A voucher specimen of *C. serratus* (ANUB No. 14096, Xiaoping Zhang) was deposited in the Herbarium Center, Anhui Normal University, China. The roots, stems and leaves of *C. serratus* were separated, crushed into coarse powders and stored at room temperature.

The coarse powders were soaked in 12 times 75% ethanol for 0.5 h, extracted for 1.5 h and then extracted with 10 times and 8 times 75% ethanol for 1 h, respectively. The tertiary filtrate was combined, concentrated under reduced pressure and dried under vacuum at 45 °C. The extraction rates of the ethanol extracts of the roots (ER), stems (ES) and leaves (EL), which were calculated as extraction rate (%) = 75% ethanol extract weight (g)/coarse powder weight (g) × 100%, were 15.35%, 11.90% and 4.31%, respectively.

The sample solutions were obtained by dissolving 0.05 g of the ER, ES and EL in 75% ethanol to obtain a concentration of 1 mg/mL, respectively. After blank correction with 75% ethanol, the solutions were scanned by using a Hitachi U-5100 spectrophotometer (Hitachi High-Tech Science Corporation, Japan) under the following spectral conditions: data mode: Abs; scanning range: 190–500 nm; scanning speed: 400 nm/min; delay: 0 s; response: fast; sampling interval: 1.0 nm; cycle time: 1.0 min; and slit width: 5.0 nm (Sun et al. 2019).

### Acute oral toxicity study

An acute oral toxicity study of the ER, ES and EL was conducted. Each rat (four SD rats/group) was orally administered a test dose of 10.35 g/kg ER, 8.05 g/kg ES or 2.90 g/kg EL. If mortality was not observed, each extract was administered the same dose to additional two animals. Then the gross morphological changes and mortality of these animals were observed for 48 h.

### Induction of AA rats and extract treatment

Thirty-six male SD rats were randomly divided into the following six groups (six rats/group): control (Con), model (MD), positive drug (PD), ER, ES and EL groups. On the 1st day, 0.1 mL of CFA (Sigma Chemicals, St. Louis, MO, USA) was injected intradermally into the right hind paw of each rat except those in the Con group. On the 12th day, the rats were administered a booster immunization with 0.08 mL of CFA. Similarly, the rats in the Con group were injected with the same amount of physiological saline.

According to preliminary experimental results and the information on converting equivalent doses among the different species, a dose equal to 50 times the clinical dose for humans was determined to be the equivalent dose for rats (Wei et al. 2010). Specifically, the daily dose for rats (extract quality, g/kg) was calculated as 3 g × 0.018 × 5 × extraction rate (ER: 15.35%; ES: 11.90%; EL: 4.31%) × 50 (multiple), where 3 g represents the mass of the *C. serratus* roots, stems or leaves, i.e., the human daily dose, 0.018 represents the conversion factor and the number “5” converts a dose of 200 g per rat to a kilogram of body weight (Nanjing University of Traditional Chinese Medicine 2006). The doses of the ER, ES and EL were 2.07, 1.61 and 0.58 g/kg, respectively.

On the 15th day, the rats in the drug groups were intragastrically administered a suspension of the corresponding extract and 0.5% CMC-Na solution. The rats in the PD group received a suspension of *Tripterygium*-polyglycosides (35 mg/kg/d) and 0.5% CMC-Na solution. The rats in the Con and MD groups received daily administrations of the same amount of 0.5% CMC-Na solution for 14 days.

### Observation of general conditions and weight changes

The general conditions (e.g., fur, activities, dietary intake, defaecation and urination) of the rats were observed and recorded daily. During the period of administration, the body weights of the rats were measured on day 0 (the day prior to CFA injection) and on the 7th, 15th, 23rd, 28th and 29th days after modelling. The body weight on day 0 was considered as the initial weight (IW), whereas the body weights on the other days were considered as the final weights (FW*x*, *x* = 7, 15, 23 and 28). The changes in the body weight of the rats (g) were calculated as FW*x* – IW.

### Calculation of the arthritis index (AI)

On the 7th, 15th, 23rd, and 28th days after modelling, the degree of systemic joint damage in the rats was observed and evaluated according to the following criteria: (1) ear: 0 = no nodules or swelling, 1 = nodules or swelling in one ear, and 2 = nodules or swelling in both ears; (2) nose: 0 = no nodules or swelling, and 1 = nodules or swelling; (3) tail: 0 = no nodules or swelling, and 1 = nodules or swelling; and (4) foot: 0 = no nodules or swelling, 1 = nodules or swelling in one foot, 2 = nodules or swelling in two feet, 3 = nodules or swelling in three feet, and 4 = nodules or swelling in four feet. The highest score was 8 points, and the sum of the scores was considered as the AI of each rat (Chen et al. 2014).

### Calculation of primary and secondary swelling rates

The perimeter of 0.5 cm below the right ankle joint was measured with cotton thread and tape on the 0, 2nd, 11th, 15th, 23rd, and 28th days after modelling to calculate the primary swelling rate. On the 0, 11th, 15th, 23rd, and 28th days after modelling, the perimeter of 0.5 cm below the left ankle joint was measured to calculate the secondary swelling rate. The swelling rate (%) was calculated as (perimeter measured at each time – perimeter before modelling)/perimeter before modelling × 100%.

### Determination of serum indicators

On the 29th day after modelling, blood samples were collected from the medial retroorbital venous plexus into capillary tubes after anaesthetization by ether inhalation, and then centrifuged at 4 °C and 3000 rpm for 15 min to obtain serum, and the levels of TNF-α, VEGF, NO, IL-1β, IL-6, MIF-1, IFN-γ, IgG, IgM, SOD and MDA were measured by using kits according to the instructions of the specifications.

### Histopathological observation

After blood collection on the 29th day, the rats, which were still under anaesthesia, were euthanized by cervical dislocation. The ankle joints of the rats were collected. The fur, muscles and tendons were removed from the ankle joints of the rats, and the ankle joints were fixed in 10% neutral formalin solution for 48 h, washed with distilled water and then decalcified with 3% nitric acid solution. The samples were embedded in paraffin and sectioned (5 μm), and the sections were stained with haematoxylin and eosin (H&E) and observed under a light microscope by two skilled pathologists unaware of the group assignment to identify the presence of synovial hyperplasia, inflammatory cell infiltration and bone or cartilage destruction. The severity of polyarthritis was scored on a scale from 0 to 4 (0 = normal, 1 = minimal, 2 = mild, 3 = moderate, and 4 = marked) (Saleem et al. 2020).

### Immunohistochemical analysis of the ankle joints

The sections were routinely dewaxed and rehydrated. Endogenous peroxidase was inactivated with 3% H_2_O_2_ solution at room temperature for 10 min. The sections were subjected to antigen repair by enzyme digestion at room temperature for 10 min and then blocked with 5% BSA at 37 °C for 30 min. Then, the sections were incubated with the primary antibody against VEGF (1:100) overnight at 4 °C and then with the goat anti-rabbit IgG at 37 °C for 30 min. The sections were subsequently treated with the SABC reagent for 30 min at 37 °C, and then with DAB for 5 min and subsequently counterstained with haematoxylin. The degree of staining was observed under an optical microscope. The positive expression rate was obtained by using ImageJ software for quantitative analysis.

### Statistical methods

Data were analysed by using Statistical Package for the Social Sciences (SPSS) software program, version 22.0 (SPSS, Inc.) and expressed as the mean ± standard deviation (SD). One-way analysis of variance was used to evaluate the differences among the groups, and then least significant difference (LSD) test was used as the *post hoc* test. A value of *p* < 0.05 indicated a significant difference.

## Results

### UV fingerprint analysis

**Figure 1.**
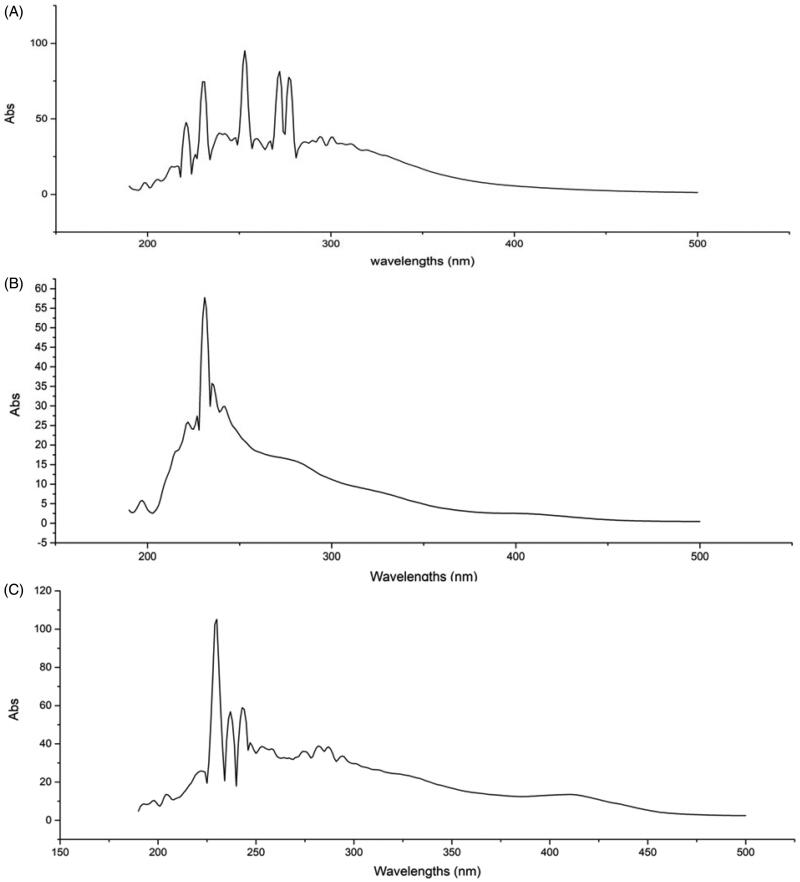
UV fingerprints of the ER (A), ES (B) and EL (C) of *C. serratus*.

As observed from the full-wavelength scanning spectra of the samples (Figure 1), the main absorption range was 200–350 nm. The ER had absorption peaks at 297, 281, 264, 249, and 234 nm; the ES had absorption peaks at 203 and 190 nm; and the EL had absorption peaks at 269, 250, and 240 nm. These findings indicated that the extracts showed absorption at characteristic wavelengths, and no interference in the range of visible light was detected.

### Acute oral toxicity analysis

The ER, ES and EL did not induce mortality at oral doses up to 10.35, 8.05 and 2.90 g/kg. The LD_50_ of the ER, ES and EL was found to be higher than 10.35, 8.05 and 2.90 g/kg, respectively. As a result, 2.07 g/kg ER, 1.61 g/kg ES and 0.58 g/kg EL were selected as the doses for the evaluation of their anti-arthritic activity in CFA-induced arthritis rats.

### Effects on the general condition of the rats

During the experiment, the activities and diets of the rats in the Con group were normal, and their fur were lustrous. In contrast, dull fur, depression, reduced diet and activities, mushy stool and obvious foot swelling were all observed in the rats after the injection of CFA. Administration of the *Tripterygium*-polyglycoside and the extracts of the different parts of *C. serratus* improved the above-mentioned symptoms of the rats to different degrees. Specifically, the rats in the PD and ER groups showed progressively lustrous fur, increased activities and diets. In contrast, the symptoms of the rats in the ES and EL groups were not evidently improved.

### Effects on the weight changes

As shown in Table 1, the body weights of all the rats increased from the 7th day to the 28th day after modelling, and the weight changes varied significantly. The CFA-induced rats showed a marked decrease in weight compared with that of the rats in the Con group (*p* < 0.01). On the 23rd and 28th days, the weight gains of the rats in the PD, ER, EL and ES groups were higher than those of the rats in the MD group. Among these treated groups, the obvious weight gains were observed in the PD, ER and EL groups (*p* < 0.01).

**Table 1.** Effects on the weight changes (mean ± SD, *n* = 6).

Group	Day 7 (g)	Day 15 (g)	Day 23 (g)	Day 28 (g)
Con	34.66 ± 3.51	71.70 ± 1.68	89.67 ± 2.52	100.00 ± 5.29
MD	26.50 ± 0.96**	59.23 ± 2.77**	72.90 ± 4.06**	76.27 ± 1.94**
PD	25.97 ± 0.74	60.93 ± 5.47	86.47 ± 2.91^##^	92.10 ± 7.48^##^
ER	26.17 ± 1.23	59.93 ± 2.40	85.30 ± 2.76^##^	92.87 ± 1.85^##^
ES	26.13 ± 0.49	60.73 ± 4.43	74.77 ± 4.80^$^	85.00 ± 2.46^#$^
EL	27.37 ± 0.55	62.17 ± 2.40	83.23 ± 3.98^##^	92.33 ± 0.98^##^

Note: *p < 0.05, **p < 0.01 *vs*. the Con group; ^#^*p* < 0.05, ^##^*p* < 0.01 *vs*. the MD group; ^$^*p* < 0.05, ^$$^*p* < 0.01 *vs*. the EL group.

### Effects on the AI

The arthritis manifestations of CFA-induced arthritis rats at different days were characterized as the AI. As presented in Table 2, an increase in the AI was observed in the rats after CFA treatment. From the 7th day to the 23rd day, the AI continuously increased in the MD group, whereas this increase was reversed after the administration of the extracts on the 15th day. Treatment with the PD, ER, and EL effectively decreased the AI from the 23rd day to the 28th day compared with that of the MD group (*p* < 0.01). The AI reduction in the ER group was more pronounced than that in the EL group on the 28th day (*p* < 0.05).

**Table 2.** Effects on the AI (mean ± SD, *n* = 6).

Group	Day 7	Day 15	Day 23	Day 28
Con	0.00 ± 0.00	0.00 ± 0.00	0.00 ± 0.00	0.00 ± 0.00
MD	2.00 ± 0.00**	3.00 ± 0.00**	3.50 ± 0.50**	3.00 ± 0.00**
PD	1.83 ± 0.41	2.50 ± 0.55	2.51 ± 0.52^##^	1.50 ± 0.55^##^
ER	1.83 ± 0.41	2.50 ± 0.55	2.50 ± 0.55^##^	1.50 ± 0.55^##$^
ES	2.17 ± 0.41	2.69 ± 0.52	3.00 ± 0.00	2.33 ± 0.52**^#^**
EL	2.00 ± 0.63	2.67 ± 0.52	2.67 ± 0.52^##^	2.00 ± 0.00^##^

Note: *p < 0.05, **p < 0.01 *vs*. the Con group; ^#^*p* < 0.05, ^##^*p* < 0.01 *vs*. the MD group; ^$^*p* < 0.05, ^$$^*p* < 0.01 *vs*. the EL group.

### Effects on the primary and secondary swelling rates

The swelling rate is an appearance indicator that reflects the severity of ankle inflammation in rats. Secondary swelling is caused by a delayed hypersensitivity reaction and usually occurs on the 11th day after modelling. As shown in Table 3, the swelling of the left posterior foot was not as obvious as that of the right posterior foot, and the secondary swelling rate reached its peak on the 23rd day.

**Table 3.** Effects on the primary swelling rate of the rats (mean ± SD, *n* = 6).

Group	2d	11d	15d	19d	28d
Con	8.45 ± 1.52	8.28 ± 1.11	8.57 ± 1.15	8.49 ± 0.28	8.84 ± 1.25
MD	28.23 ± 0.33**	19.40 ± 0.93**	20.32 ± 0.15**	28.24 ± 0.56**	22.73 ± 1.05**
PD	27.73 ± 0.27	19.22 ± 0.68	19.38 ± 0.36	19.73 ± 0.36^##^	13.20 ± 0.20^##^
ER	27.59 ± 0.47	19.80 ± 0.18	19.44 ± 0.50	19.46 ± .022^##^^$$^	16.19 ± 0.53^##^^$$^
ES	27.42 ± 0.93	18.73 ± 1.19	19.45 ± 0.12	22.63 ± 0.52^##^	21.53 ± 0.13^$$^
EL	28.43 ± 0.60	19.74 ± 0.44	19.40 ± 0.15	22.07 ± 0.70^##^	18.32 ± 0.16^##^

Note: *p < 0.05, **p < 0.01 *vs*. the Con group; *^#^p* < 0.05, ^##^*p* < 0.01 *vs*. the MD group; ^$^*p* < 0.05, ^$$^*p* < 0.01 *vs*. the EL group.

CFA induced significant swelling in the hind paw of the rats (*p* < 0.01). Treatment with the *Tripterygium*-polyglycoside and different extracts of *C. serratus* notably reduced the primary and secondary swelling rates of the rats from the 23rd day to the 28th day compared with the MD group (*p* < 0.01 or *p* < 0.05). Compared with those of the EL group, the primary and secondary swelling rates of the PD and ER groups were significantly decreased on the 28th day (*p* < 0.01 or *p* < 0.05), whereas the primary and secondary swelling rates of the ES group were clearly increased (*p* < 0.05), as shown in Table 4.

**Table 4.** Effects on the secondary swelling rate of the rats (mean ± SD, *n* = 6).

Group	11d	15d	23d	28d
Con	4.53 ± 0.36	4.55 ± 0.24	4.56 ± 0.30	4.52 ± 0.19
MD	16.32 ± 0.06**	17.13 ± 0.17**	26.50 ± 0.80**	22.74 ± 1.19**
PD	16.26 ± 0.05	16.82 ± 0.14	16.98 ± 0.30^##^	12.98 ± 0.73^##^
ER	16.42 ± 0.06	16.75 ± 0.22	16.80 ± 0.21^##^	15.85 ± 0.39^##^^$^
ES	16.29 ± 0.07	17.51 ± 0.38	22.39 ± 0.34^##^^$$^	21.29 ± 0.56^#^^$$^
EL	16.38 ± 0.04	17.07 ± 0.50	17.65 ± 0.63^##^	17.14 ± 0.30^##^

Note: *p < 0.05, **p < 0.01 *vs*. the Con group; *^#^p* < 0.05, ^##^*p* < 0.01 *vs*. the MD group; ^$^*p* < 0.05, ^$$^*p* < 0.01 *vs*. the EL group.

### Effects on serum TNF-α and VEGF levels

**Figure 2.**
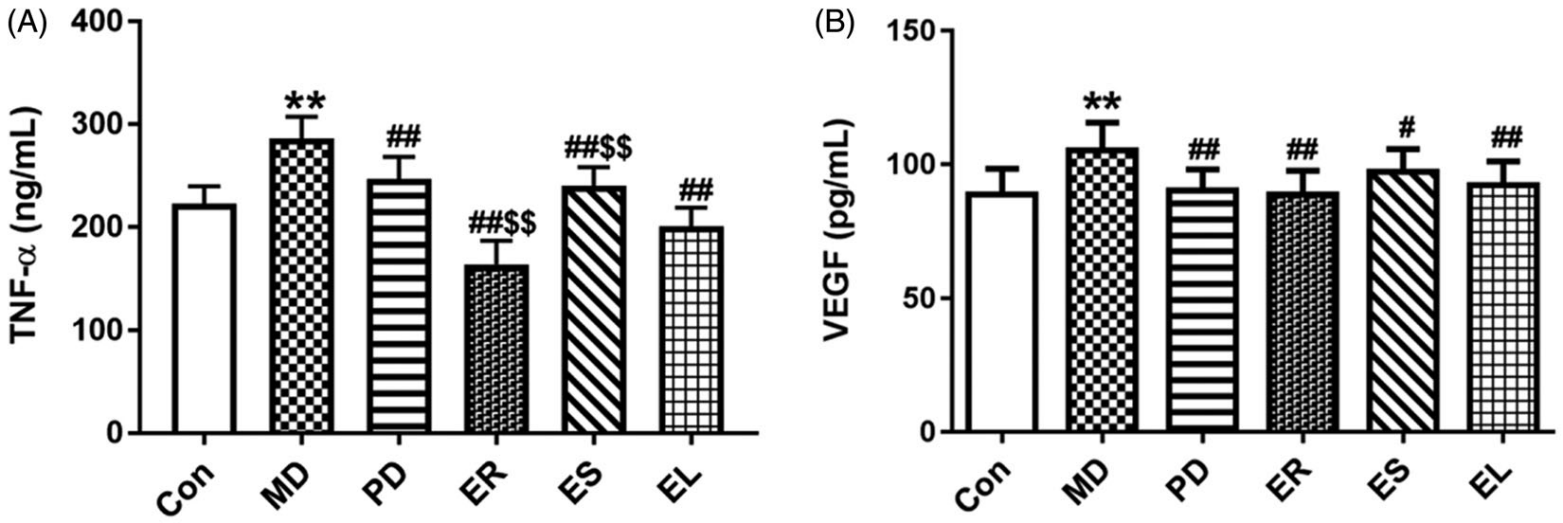
Effects on the serum levels of TNF-α (A) and VEGF (B). The rats were treated with the ER (2.07 g/kg/d), ES (1.61 g/kg/d), EL (0.58 g/kg/d) and *Tripterygium*-polyglycosides (35 mg/kg/d) for 14 consecutive days after modelling. After administration, the levels of TNF-α and VEGF in each extract group were decreased to different extents, among them, the ER group was the most significant, followed by the EL group. All data were represented as mean ± SD (*n* = 6). *p < 0.05, **p < 0.01 *vs*. the Con group; *^#^p* < 0.05, ^##^*p* < 0.01 *vs*. the MD group; ^$^*p* < 0.05, ^$$^*p* < 0.01 *vs*. the EL group.

Substantial increases in the levels of TNF-α and VEGF were observed in CFA-treated rats compared with the rats in the Con group (*p* < 0.01). In contrast to the MD group, marked decreases were detected in the PD and extract-treated groups (*p* < 0.05, *p* < 0.01), particularly the ER group. And the TNF-α level in the ER group was significantly decreased (*p* < 0.01) compared with that in the EL group, and the opposite result was observed in the ES group (*p* < 0.01, Figure 2).

### Effects on serum NO, IL-1β, IL-6 and MIF-1 levels

**Figure 3.**
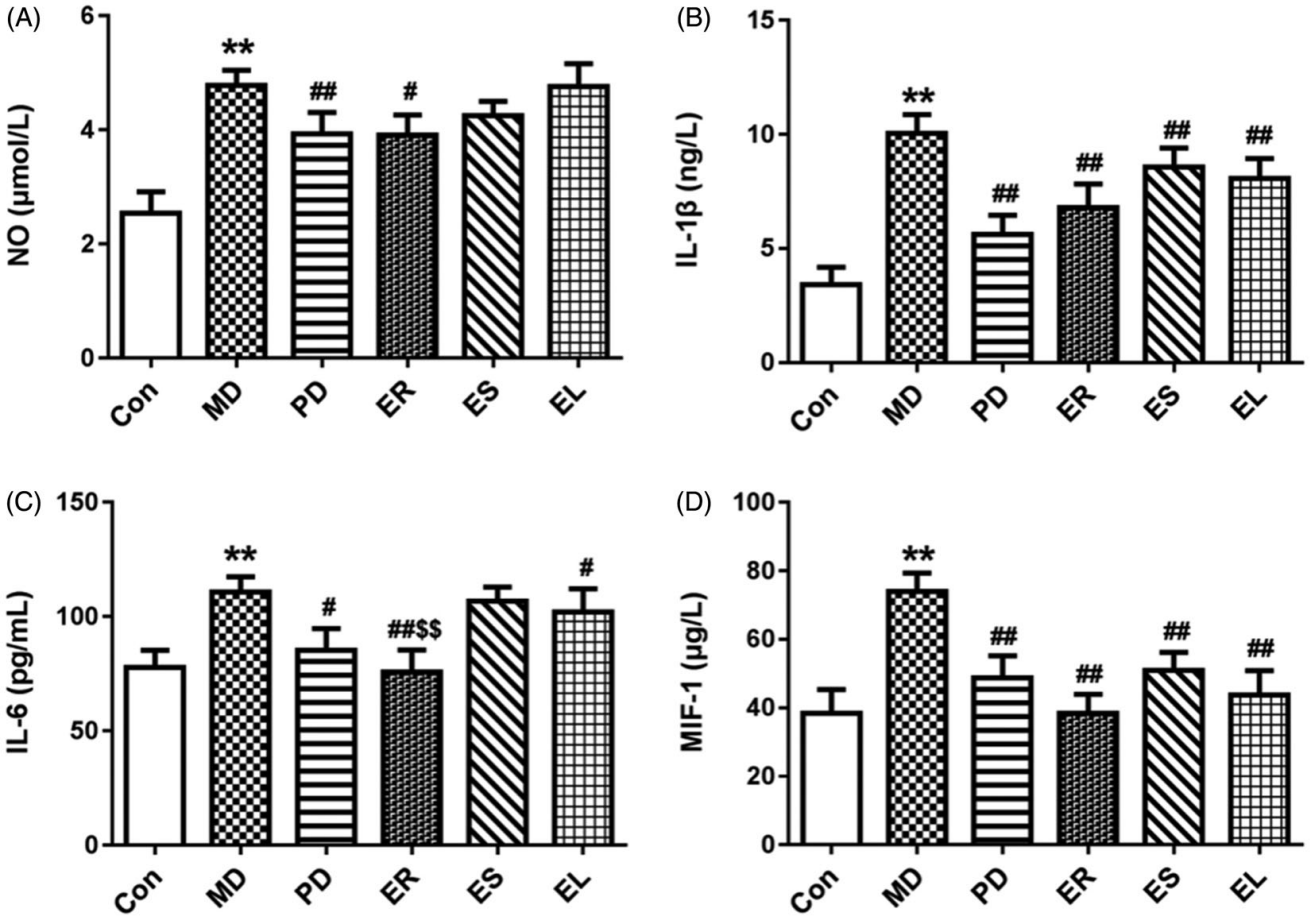
Effects on the levels of NO (A), IL-1β (B), IL-6 (C) and MIF-1 (D). The rats were treated with the ER (2.07 g/kg/d), ES (1.61 g/kg/d), EL (0.58 g/kg/d) and *Tripterygium*-polyglycosides (35 mg/kg/d) for 14 consecutive days after modelling. After administration, the expression of NO, IL-1β, IL-6 and MIF-1 in each extract group was decreased to different extents, and the ER group was the most obvious, followed by the EL group. All data were represented as mean ± SD (*n* = 6). *p < 0.05, **p < 0.01 *vs*. the Con group; ^#^*p* < 0.05, ^##^*p* < 0.01 *vs*. the MD group; ^$^*p* < 0.05, ^$$^*p* < 0.01 *vs*. the EL group.

Considerable increases (*p* < 0.01) in the levels of NO, IL-1β, IL-6 and MIF-1 were observed after CFA treatment, as shown in Figure 3. However, treatment with the *Tripterygium*-polyglycoside and plant extracts reduced these increases in the arthritic rats. Among the extracts, the ER induced the most prominent inhibition (*p* < 0.05 or *p* < 0.01), followed by the ES and EL.

### Effects on the levels of IFN-γ, IgG and IgM

**Figure 4.**
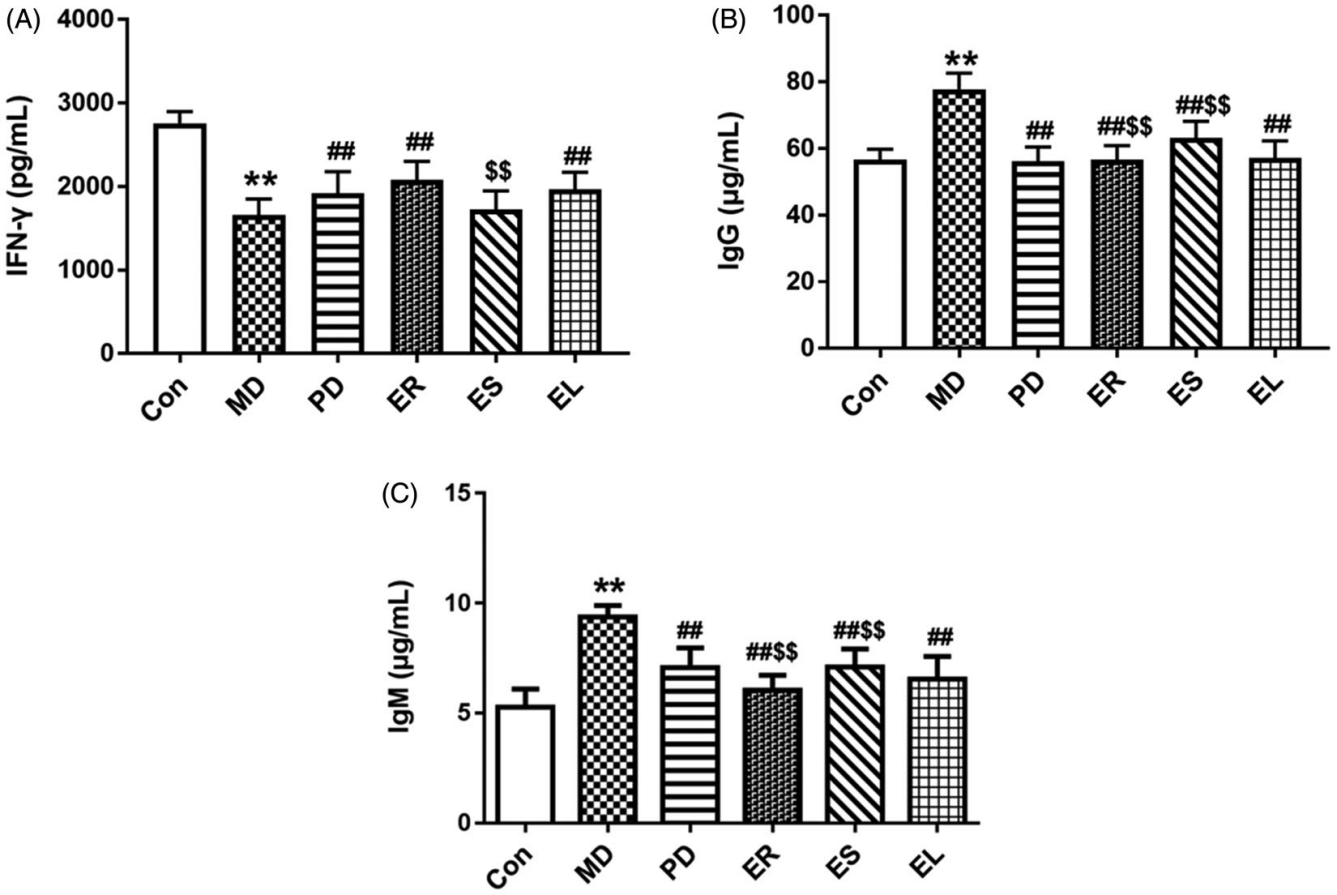
Effects on the levels of IFN-γ (A), IgG (B) and IgM (C). The rats were treated with the ER (2.07 g/kg/d), ES (1.61 g/kg/d), EL (0.58 g/kg/d) and *Tripterygium*-polyglycosides (35 mg/kg/d) for 14 consecutive days after modelling. After treating with the different extracts, the level of IFN-γ was increased, whereas the levels of IgG and IgM were decreased compared with those of the MD group. Moreover, the ER group was the most obvious, followed by the EL group. All data were represented as mean ± SD (*n* = 6). *p < 0.05, **p < 0.01 *vs*. the Con group; ^#^*p* < 0.05, ^##^*p* < 0.01 *vs*. the MD group; ^$^*p* < 0.05, ^$$^*p* < 0.01 *vs*. the EL group.

The rats with CFA-induced arthritis showed decreased IFN-γ level and increased IgG and IgM levels (*p* < 0.01, Figure 4), and these changes were reversed by treatment with the PD and different extracts, particularly the ER (*p* < 0.01). Compared with those in the EL group, the levels of IgG and IgM in the ER group were significantly decreased (*p* < 0.01), whereas clearly increased in the ES group (*p* < 0.01).

### Effects on serum SOD and MDA levels

**Figure 5.**
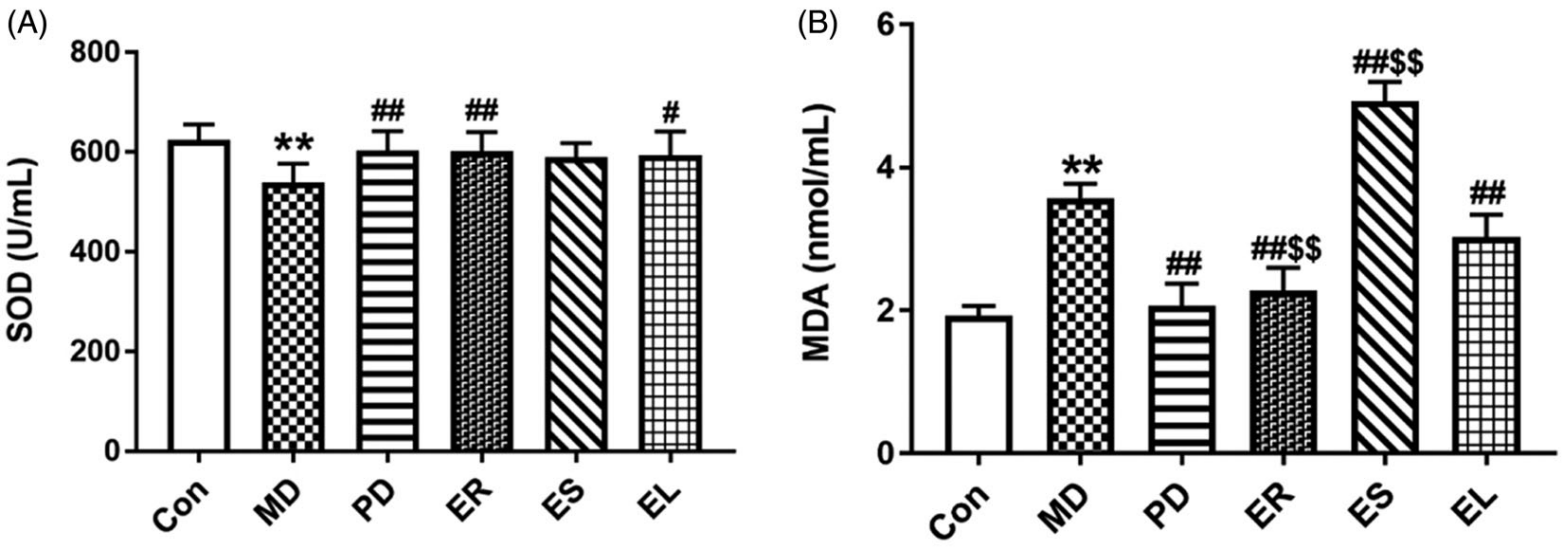
Effects on the levels of SOD (A) and MDA (B). The rats were treated with the ER (2.07 g/kg/d), ES (1.61 g/kg/d), EL (0.58 g/kg/d) and *Tripterygium*-polyglycosides (35 mg/kg/d) for 14 consecutive days after modelling. Compared with the MD group, the level of SOD increased under the treatment of the different extracts in all the groups. Except for the ES group, the level of MDA decreased in all the groups. All data were represented as mean ± SD (*n* = 6). *p < 0.05, **p < 0.01 *vs*. the Con group; ^#^*p* < 0.05, ^##^*p* < 0.01 *vs*. the MD group; ^$^*p* < 0.05, ^$$^*p* < 0.01 *vs*. the EL group.

CFA treatment substantially reduced the level of SOD and increased the level of MDA (*p* < 0.01). As shown in Figure 5, treatment with the PD, ER, and ES increased the SOD level and markedly decreased the MDA level. The increase in SOD level treated by the ES was not significant (*p* > 0.05). Additionally, treatment with the ES did not reverse but aggravated CFA-induced increase in MDA level. The level of MDA was markedly decreased (*p* < 0.01) in the ER group, but clearly increased in the ES group (*p* < 0.01) compared with that in the EL group.

### Histopathological analysis

**Figure 6.**
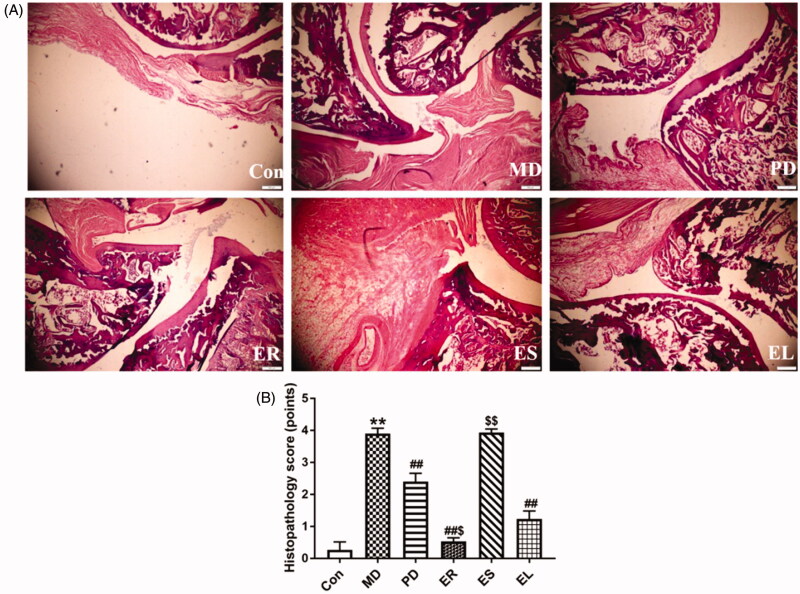
Histopathological effects of the different part extracts of *C. serratus* on CFA-induced arthritic rats (HE, × 200). The rats were treated with the ER (2.07 g/kg/d), ES (1.61 g/kg/d), EL (0.58 g/kg/d) and *Tripterygium*-polyglycosides (35 mg/kg/d) for 14 consecutive days after modelling. (A) Representative photos of HE staining. (B) Histopathology score (points). The damage was the most serious in the ES group, followed by the EL group. All data were represented as mean ± SD (*n* = 6). *p < 0.05, **p < 0.01 *vs*. the Con group; *^#^p* < 0.05, ^##^*p* < 0.01 *vs*. the MD group; ^$^*p* < 0.05, ^$$^*p* < 0.01 *vs*. the EL group.

As shown in Figure 6, the rats in the Con group did not show pathological changes. The histopathology analysis of the ankle joint revealed the occurrence of tissue damage in the rats with CFA-induced arthritis, as indicated by a narrow joint cavity, an unclear and rough surface, a convex synovial villus and proliferated synovial cells, as well as inflammatory exudation. Although synovial cell proliferation, bone tissue damage and a small amount of inflammatory cell infiltration were still observed, the degree of the inflammation was significantly alleviated when treated with the PD, ER and EL, particularly the ER compared with that in the MD group. Nonetheless, the degree of synovial hyperplasia in the ES group remained severe.

### Effects on the positive expression of VEGF in ankle joint synovial tissues

**Figure 7.**
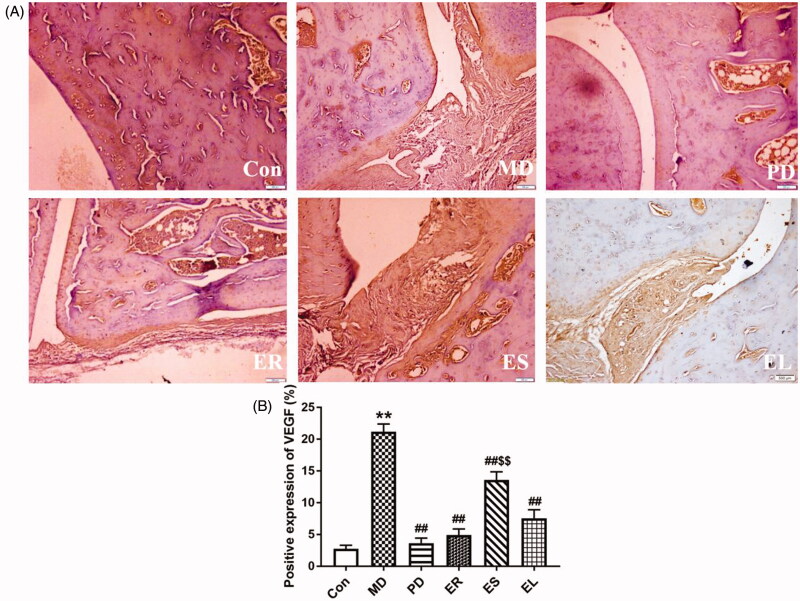
Effects on the positive expression of VEGF (× 200). The rats were treated with the ER (2.07 g/kg/d), ES (1.61 g/kg/d), EL (0.58 g/kg/d) and *Tripterygium*- polyglycosides (35 mg/kg/d) for 14 consecutive days after modelling. (A) Immunohistochemistry results of VEGF. (B) Column chart represented the positive expression rate of VEGF. All data were represented as mean ± SD (*n* = 6). *p < 0.05, **p < 0.01 *vs*. the Con group; ^#^*p* < 0.05, ^##^*p* < 0.01 *vs*. the MD group; ^$^*p* < 0.05, ^$$^*p* < 0.01 *vs*. the EL group.

The positive expression of VEGF is indicated by the number of tan particles around the synovium of the rats. As presented in Figure 7, the injection of CFA significantly increased the positive expression rate of VEGF compared with that of the Con group (*p* < 0.01). This study revealed that the PD and all the extracts markedly decreased the positive expression rate of VEGF compared with that in the MD group (*p* < 0.01). And the positive expression rate of VEGF was significantly decreased in the ER group (*p* < 0.05), whereas markedly increased in the ES group (*p* < 0.05) compared with that in the EL group.

## Discussion

RA, a common progressive autoimmune disease, is characterized by the interactions of various inflammatory mediators. This disease has complex causes and multiple pathogenic mechanisms (Al-Herz et al. 2017). Although the aetiology remains unclear, researchers have recognized that autoimmunity is active and inflammatory chemokines are abnormally aggravated in patients with RA. Therefore, the anti-arthritic effects of the extracts from different parts of *C. serratus* were clarified by the determination of inflammatory mediators.

The AA rat model is an acute model induced by CFA (Tuncel et al. 2016). Its clinical manifestations, serological indicators and pathological changes are similar to those observed in human RA in many aspects, so the AA rat model has been widely used in studies of RA and the screening of anti-inflammatory drugs (Li et al. 2008).

In the presence of inflammation, macrophages secrete pro-inflammatory cytokines, such as TNF-α and VEGF, which are highly expressed in the synovial tissues and synovial fluid of RA patients (Costa et al. 2016). TNF-α promotes the formation and growth of synovitis and pannus, and invades tissues to destroy cartilage and bone during the activation of RA (Tung et al. 2017). The secretion of TNF-α induces the secretion of IL-1β, and they play a synergistic role in the pathogenesis of RA (Bonetti et al. 2019). TNF-α also upregulates the expression of VEGF in the lesions (Costa et al. 2016). VEGF, a marker of bone destruction, increases microvascular hyperpermeability, induces endothelial cell proliferation and inflammatory cell infiltration, promotes the formation of new blood vessels and leads to continuous destruction of articular cartilage in the pathogenesis of RA (Gudbjörnsson et al. 2004).

The complex cytokines and chemokines released from the synovium are critical to the pathophysiology of RA. High level of NO causes damage to synovial tissues, vasodilation and inflammatory exudation (Tu et al. 2019). MIF-1 stimulates the releases of TNF-α, IL-1β and IL-6, promotes angiogenesis and leads to synovial cell proliferation, which is greatly increased in synovial fluid and synovial tissues of patients with RA (Zhang et al. 2018). And inhibiting the level of MIF-1 can control inflammation and cartilage destruction (Li et al. 2008). IL-6 induces neovascularization to form pannus, which leads to synovial hyperplasia and inflammatory cell infiltration and aggravates articular cartilage tissue lesions, and it is an effective target for interventions in inflammatory diseases (Hirota et al. 2016; Kamel et al. 2018).

In this study, the administration of all tested extracts reduced the TNF-α, NO, IL-1β, IL-6, VEGF and MIF-1 levels and the positive expression rate of VEGF to varying degrees, and among the extracts, the ER exerted the most significant effect, followed by the EL.

IFN-γ, one of the most important innate immunomodulators in the host, has a potential therapeutic effect on RA (Schurgers et al. 2012). Rheumatoid factor (RF) and IgG form an immune complex that activates the complement system, thereby inducing inflammatory responses (Elberry et al. 2010). RF-IgM is the only serological indicator recognized by the American Rheumatology Association for the classification and diagnosis of RA (Hjeltnes et al. 2011), and the levels of IgG and IgM are increased in the serum of RA patients (Gińdzieńska-Sieśkiewicz et al. 2016). In this study, the administration of the different extracts increased the IFN-γ level and decreased the levels of IgG and IgM. The effects of the ER were the most obvious, followed by the EL.

SOD, an endogenous antioxidant enzyme, scavenges free radicals and reduces the occurrence of lipid peroxidation (Grönwall et al. 2017). MDA, one of the lipid peroxidation products, indirectly reflects the severity of free radical attacking on cells (He et al. 2006). In this study, the increase in the MDA content and the decrease in the SOD content induced by CFA were markedly restored by treatment with the ER, indicating that the ER had certain anti-inflammatory activity, and it was associated with increased antioxidant capacity.

Histopathological investigations also confirmed the anti-arthritis effects, which were consistent with the results of inflammatory mediators. The administration of each ethanol extract ameliorated inflammatory cell infiltration, synovial hyperplasia and articular cartilage destruction, and the most obvious improvements were obtained with the ER, followed by the EL. Reducing the swelling rates, AI and organ damage and reversing the weight changes in CFA-induced arthritis rats also supported the above anti-inflammatory effects.

Taken together, these findings provide evidence for the anti-arthritic activity of different parts of *C. serratus* in CFA-induced arthritis rats, which is related to inhibition of the releases of inflammatory cytokines and improving antioxidant capacity. The results also show that the ER is the most efficient, followed by the EL. Our future research directions will involve the development of *C. serratus* as a new anti-RA drug and its clinical applications.

## Data Availability

All the data generated or analysed during this study are included in this published article.
